# Crystal structure of 2-(4-chloro­phen­yl)-4-(1*H*-indol-3-yl)-6-phenyl­pyridine-3-carbo­nitrile

**DOI:** 10.1107/S1600536814017693

**Published:** 2014-08-06

**Authors:** R. Vishnupriya, J. Suresh, Pethaiah Gunasekaran, Subbu Perumal, P. L. Nilantha Lakshman

**Affiliations:** a Department of Physics, The Madura College, Madurai 625 011, India; b Department of Organic Chemistry, School of Chemistry, Madurai Kamaraj University, Madurai 625 021, India; c Department of Food Science and Technology, University of Ruhuna, Mapalana, Kamburupitiya 81100, Sri Lanka

**Keywords:** crystal structure, pyridine-3-carbo­nitrile, hydrogen bonding

## Abstract

In the title compound, C_26_H_16_ClN_3_, the dihedral angles between the central pyridine ring and the pendant phenyl, chloro­benzene and indole rings are 18.52 (12), 48.97 (11) and 21.20 (10)°, respectively. An intra­molecular C—H⋯N_c_ (c = cyanide) hydrogen bond occurs. In the crystal, inversion dimers linked by pairs of N—H⋯N_c_ hydrogen bonds generate *R*_2_^2^(16) loops.

## Related literature   

For the biological activity of substituted pyridine derivatives, see: Yao *et al.* (1994 ▶). For a related structure, see: Vishnupriya *et al.* (2014 ▶).
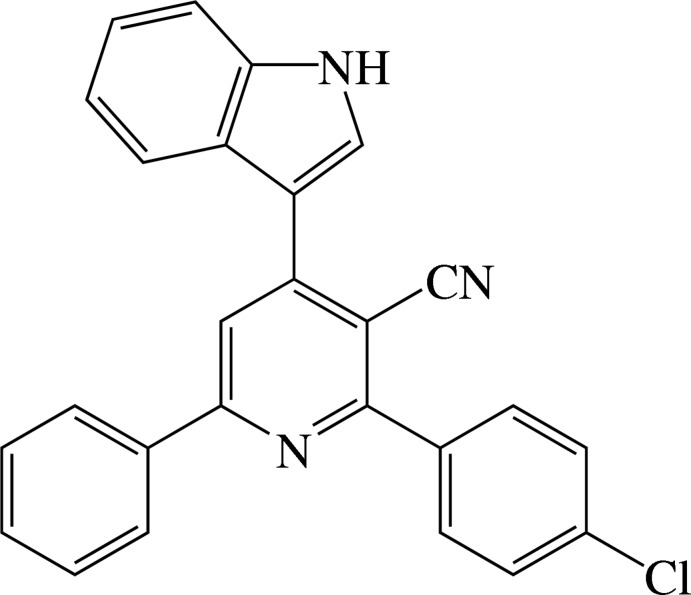


## Experimental   

### Crystal data  

C_26_H_16_ClN_3_
*M* *_r_* = 405.87
Monoclinic, 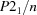

*a* = 7.6533 (4) Å
*b* = 11.4822 (7) Å
*c* = 23.2906 (14) Å
β = 94.351 (1)°
*V* = 2040.8 (2) Å^3^
*Z* = 4
Mo *K*α radiation
μ = 0.21 mm^−1^
*T* = 293 K
0.50 × 0.25 × 0.20 mm

### Data collection  

Bruker Kappa APEXII CCD diffractometer
Absorption correction: multi-scan (*SADABS*; Bruker, 2004 ▶) *T*_min_ = 0.958, *T*_max_ = 0.986
15210 measured reflections
4194 independent reflections
3000 reflections with *I* > 2σ(*I*)
*R*_int_ = 0.028

### Refinement  

*R*[*F* ^2^ > 2σ(*F* ^2^)] = 0.051
*wR*(*F* ^2^) = 0.146
*S* = 1.04
4194 reflections
271 parameters
H-atom parameters constrained
Δρ_max_ = 0.38 e Å^−3^
Δρ_min_ = −0.40 e Å^−3^

### 

Data collection: *APEX2* (Bruker, 2004 ▶); cell refinement: *SAINT* (Bruker, 2004 ▶); data reduction: *SAINT*; program(s) used to solve structure: *SHELXS97* (Sheldrick, 2008 ▶); program(s) used to refine structure: *SHELXL97* (Sheldrick, 2008 ▶); molecular graphics: *PLATON* (Spek, 2009 ▶); software used to prepare material for publication: *SHELXL97*.

## Supplementary Material

Crystal structure: contains datablock(s) global, I. DOI: 10.1107/S1600536814017693/hb7263sup1.cif

Structure factors: contains datablock(s) I. DOI: 10.1107/S1600536814017693/hb7263Isup2.hkl

Supporting information file. DOI: 10.1107/S1600536814017693/hb7263Isup3.cml

**Fig. 1.**
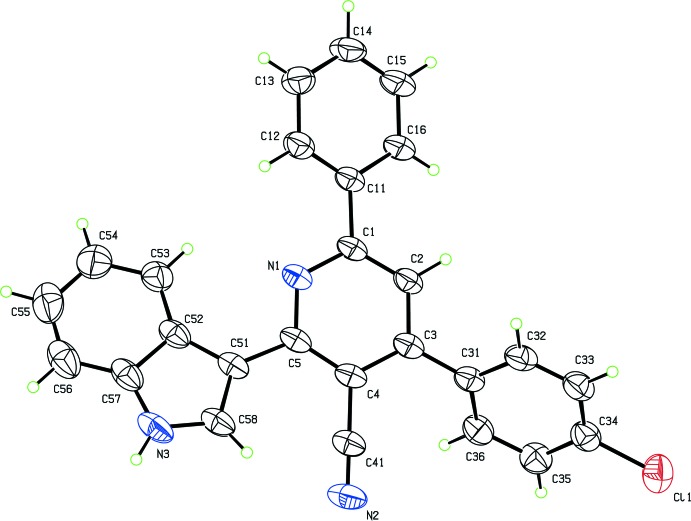
The molecular structure of compound showing 30% probability displacement ellipsoids.

. DOI: 10.1107/S1600536814017693/hb7263fig1.tif
The mol­ecular structure of compound showing 30% probability displacement ellipsoids.

**Fig. 2.**
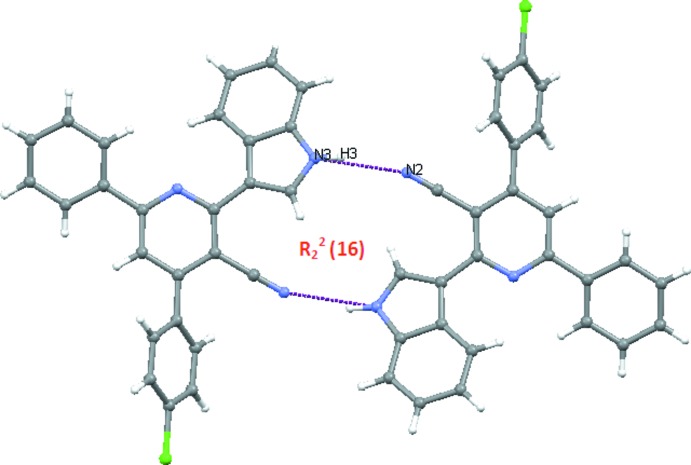
Partial packing view of the compound showing molecules linked by a pair of N—H···N interactions (dotted lines).

. DOI: 10.1107/S1600536814017693/hb7263fig2.tif
Partial packing view of the compound showing mol­ecules linked by a pair of N—H⋯N inter­actions (dotted lines).

CCDC reference: 1017501

Additional supporting information:  crystallographic information; 3D view; checkCIF report

## Figures and Tables

**Table 1:** Hydrogen-bond geometry (Å, °)

*D*—H⋯*A*	*D*—H	H⋯*A*	*D*⋯*A*	*D*—H⋯*A*
C58—H58⋯N2	0.93	2.58	3.285 (4)	133
N3—H3⋯N2^i^	0.86	2.20	3.037 (3)	164

Symmetry code: (i) 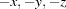.

## supplementary crystallographic information

### S1. Comment

Thienopyridines have been used as antithrombotic agents (Yao *et al.*, 1994) against platelet aggregation. As part of our ongoing studies in this area (Vishnupriya *et al.*, 2014) the title compound was investigated.

The deviation of the nitrile atoms (C41,N2) from the mean plane of the pyridine ring system is 0.0579 (5) and 0.0826 (2) Å. The shortening of the C—N distances [1.340 (3) and 1.348 (3) Å] and the opening of the N1–C4–C5 angle [120.83 (2)°] may be attributed to the size of the substituent at C1, correlating well with the values observed in the *ortho*-substituted derivative. The carbonitrile group lies almost in the plane of the attached planar pyridine ring system.

The crystal structure features a N3—H3···N2^(i)^ [symmetry code: (i) 2 - *x*, 2 - *y*, -*z*] hydrogen bonded *R*_2_^2^ (16) motif (Fig. 2). No significant π—π stacking interaction between neighboring aromatic rings or C—H···π interaction towards them are observed.

### S2. Experimental

A mixture of 3-(1*H*-indol-3-yl)-3-oxopropanenitrile 1 (1 mmol), 4,4,4-trifluoro-1-phenylbutane-1,3-dione 2 (1 mmol) and 4-chloro benzaldehyde 3 (1 mmol) in the presence of ammonium acetate (400 mmol) under solvent-free condition was heated at 110 °C for 6 h. After completion of the reaction (TLC), the reaction mixture was poured into water and extracted with dichloromethane. After removal of the solvent, the residue was chromatographed over silica gel (230–400 mesh) using petroleum ether-ethyl acetate mixture (7:3 *v*/*v*), which afforded the pure compound. Melting point:273 °C, Yield: 70%.

### S3. Refinement

H atoms were placed at calculated positions and allowed to ride on their carrier atoms with C—H = 0.93–0.98 Å and with *U*_iso_ = 1.2*U*_eq_(C, N) for N, CH_2_ and CH atoms and *U*_iso_ = 1.5*U*_eq_(C) for CH_3_ atoms.

### Crystal data

**Table:**

C_26_H_16_ClN_3_	*F*(000) = 840
*M**_r_* = 405.87	*D*_x_ = 1.321 Mg m^−^^3^
Monoclinic, *P*2_1_/*n*	Mo *K*α radiation, λ = 0.71073 Å
Hall symbol: -P 2yn	Cell parameters from 2000 reflections
*a* = 7.6533 (4) Å	θ = 2–27°
*b* = 11.4822 (7) Å	µ = 0.21 mm^−^^1^
*c* = 23.2906 (14) Å	*T* = 293 K
β = 94.351 (1)°	Block, colourless
*V* = 2040.8 (2) Å^3^	0.50 × 0.25 × 0.20 mm
*Z* = 4	

### Data collection

**Table:**

Bruker Kappa APEXII CCD diffractometer	4194 independent reflections
Radiation source: fine-focus sealed tube	3000 reflections with *I* > 2σ(*I*)
Graphite monochromator	*R*_int_ = 0.028
Detector resolution: 0 pixels mm^-1^	θ_max_ = 26.5°, θ_min_ = 1.8°
ω and φ scans	*h* = −9→9
Absorption correction: multi-scan (*SADABS*; Bruker, 2004)	*k* = −14→13
*T*_min_ = 0.958, *T*_max_ = 0.986	*l* = −29→19
15210 measured reflections	

### Refinement

**Table:**

Refinement on *F*^2^	Primary atom site location: structure-invariant direct methods
Least-squares matrix: full	Secondary atom site location: difference Fourier map
*R*[*F*^2^ > 2σ(*F*^2^)] = 0.051	Hydrogen site location: inferred from neighbouring sites
*wR*(*F*^2^) = 0.146	H-atom parameters constrained
*S* = 1.04	*w* = 1/[σ^2^(*F*_o_^2^) + (0.054*P*)^2^ + 0.8601*P*] where *P* = (*F*_o_^2^ + 2*F*_c_^2^)/3
4194 reflections	(Δ/σ)_max_ < 0.001
271 parameters	Δρ_max_ = 0.38 e Å^−^^3^
0 restraints	Δρ_min_ = −0.40 e Å^−^^3^

### Special details

**Table:**

Geometry. All e.s.d.'s (except the e.s.d. in the dihedral angle between two l.s. planes) are estimated using the full covariance matrix. The cell e.s.d.'s are taken into account individually in the estimation of e.s.d.'s in distances, angles and torsion angles; correlations between e.s.d.'s in cell parameters are only used when they are defined by crystal symmetry. An approximate (isotropic) treatment of cell e.s.d.'s is used for estimating e.s.d.'s involving l.s. planes.
Refinement. Refinement of *F*^2^ against ALL reflections. The weighted *R*-factor *wR* and goodness of fit *S* are based on *F*^2^, conventional *R*-factors *R* are based on *F*, with *F* set to zero for negative *F*^2^. The threshold expression of *F*^2^ > σ(*F*^2^) is used only for calculating *R*-factors(gt) *etc*. and is not relevant to the choice of reflections for refinement. *R*-factors based on *F*^2^ are statistically about twice as large as those based on *F*, and *R*- factors based on ALL data will be even larger.

### Fractional atomic coordinates and isotropic or equivalent isotropic displacement parameters (Å^2^)

**Table:**

	*x*	*y*	*z*	*U*_iso_*/*U*_eq_	
C1	0.2617 (3)	0.54731 (16)	−0.00655 (10)	0.0574 (5)	
C2	0.2444 (3)	0.53305 (18)	−0.06568 (10)	0.0627 (5)	
H2	0.2589	0.5966	−0.0896	0.075*	
C3	0.2057 (3)	0.42467 (17)	−0.08943 (10)	0.0603 (5)	
C4	0.1803 (3)	0.33296 (17)	−0.05109 (10)	0.0605 (5)	
C5	0.1900 (3)	0.35418 (17)	0.00869 (10)	0.0598 (5)	
C11	0.3096 (3)	0.66136 (17)	0.02012 (10)	0.0580 (5)	
C12	0.2778 (3)	0.6854 (2)	0.07626 (11)	0.0704 (6)	
H12	0.2292	0.6283	0.0985	0.085*	
C13	0.3173 (4)	0.7936 (2)	0.10002 (12)	0.0806 (7)	
H13	0.2941	0.8091	0.1379	0.097*	
C14	0.3902 (4)	0.8774 (2)	0.06809 (14)	0.0895 (8)	
H14	0.4130	0.9510	0.0835	0.107*	
C15	0.4297 (5)	0.8525 (2)	0.01292 (14)	0.1014 (10)	
H15	0.4846	0.9085	−0.0083	0.122*	
C16	0.3893 (4)	0.74587 (19)	−0.01150 (12)	0.0785 (7)	
H16	0.4153	0.7305	−0.0491	0.094*	
C31	0.1913 (3)	0.41209 (18)	−0.15290 (10)	0.0613 (5)	
C32	0.0952 (3)	0.49362 (19)	−0.18691 (11)	0.0721 (6)	
H32	0.0370	0.5533	−0.1692	0.087*	
C33	0.0853 (3)	0.4870 (2)	−0.24595 (12)	0.0762 (7)	
H33	0.0199	0.5413	−0.2680	0.091*	
C34	0.1727 (3)	0.3996 (2)	−0.27214 (11)	0.0714 (6)	
C35	0.2690 (3)	0.3182 (2)	−0.23988 (12)	0.0763 (7)	
H35	0.3275	0.2592	−0.2579	0.092*	
C36	0.2779 (3)	0.3248 (2)	−0.18073 (11)	0.0707 (6)	
H36	0.3430	0.2698	−0.1590	0.085*	
C41	0.1423 (3)	0.21813 (19)	−0.07380 (11)	0.0692 (6)	
C51	0.1586 (3)	0.26723 (17)	0.05255 (11)	0.0630 (6)	
C52	0.2163 (3)	0.27513 (18)	0.11280 (11)	0.0674 (6)	
C53	0.3179 (3)	0.3535 (2)	0.14722 (12)	0.0778 (7)	
H53	0.3638	0.4198	0.1311	0.093*	
C54	0.3493 (4)	0.3314 (3)	0.20519 (13)	0.0965 (9)	
H54	0.4174	0.3830	0.2281	0.116*	
C55	0.2800 (5)	0.2320 (3)	0.23029 (15)	0.1080 (11)	
H55	0.3005	0.2198	0.2697	0.130*	
C56	0.1834 (5)	0.1534 (3)	0.19779 (15)	0.1012 (10)	
H56	0.1402	0.0867	0.2144	0.121*	
C57	0.1506 (4)	0.1750 (2)	0.13923 (13)	0.0793 (8)	
C58	0.0637 (3)	0.16548 (19)	0.04622 (12)	0.0762 (7)	
H58	0.0101	0.1382	0.0116	0.091*	
N1	0.2322 (2)	0.46053 (14)	0.02994 (8)	0.0597 (4)	
N2	0.1113 (3)	0.12741 (17)	−0.09152 (11)	0.0913 (7)	
N3	0.0600 (3)	0.11130 (17)	0.09728 (11)	0.0870 (7)	
H3	0.0083	0.0462	0.1027	0.104*	
Cl1	0.15880 (11)	0.38830 (8)	−0.34663 (3)	0.0995 (3)	

### Atomic displacement parameters (Å^2^)

**Table:**

	*U*^11^	*U*^22^	*U*^33^	*U*^12^	*U*^13^	*U*^23^
C1	0.0505 (11)	0.0401 (10)	0.0811 (15)	−0.0067 (8)	0.0018 (10)	0.0034 (10)
C2	0.0666 (13)	0.0441 (10)	0.0768 (15)	−0.0091 (9)	0.0018 (11)	0.0061 (10)
C3	0.0532 (11)	0.0453 (10)	0.0819 (15)	−0.0039 (9)	0.0027 (10)	0.0022 (10)
C4	0.0544 (12)	0.0409 (10)	0.0863 (16)	−0.0055 (8)	0.0065 (10)	−0.0016 (10)
C5	0.0513 (11)	0.0407 (10)	0.0880 (16)	−0.0045 (8)	0.0090 (10)	0.0050 (10)
C11	0.0540 (11)	0.0424 (10)	0.0765 (14)	−0.0055 (8)	−0.0023 (10)	0.0028 (10)
C12	0.0736 (15)	0.0545 (12)	0.0825 (16)	−0.0103 (11)	0.0020 (12)	0.0030 (11)
C13	0.0880 (18)	0.0691 (15)	0.0830 (17)	−0.0071 (13)	−0.0048 (13)	−0.0115 (13)
C14	0.107 (2)	0.0522 (13)	0.107 (2)	−0.0207 (13)	−0.0058 (17)	−0.0129 (14)
C15	0.143 (3)	0.0541 (14)	0.108 (2)	−0.0385 (16)	0.016 (2)	−0.0021 (15)
C16	0.1008 (19)	0.0495 (12)	0.0855 (17)	−0.0226 (12)	0.0087 (14)	−0.0010 (11)
C31	0.0568 (12)	0.0474 (11)	0.0789 (15)	−0.0062 (9)	0.0001 (10)	−0.0001 (10)
C32	0.0752 (15)	0.0486 (11)	0.0911 (18)	0.0061 (11)	−0.0034 (13)	−0.0035 (11)
C33	0.0784 (16)	0.0579 (13)	0.0899 (18)	0.0005 (12)	−0.0088 (13)	0.0089 (13)
C34	0.0670 (14)	0.0670 (14)	0.0799 (16)	−0.0088 (11)	0.0040 (12)	0.0073 (12)
C35	0.0735 (15)	0.0711 (15)	0.0862 (18)	0.0094 (12)	0.0172 (13)	0.0007 (13)
C36	0.0646 (14)	0.0624 (13)	0.0848 (17)	0.0105 (11)	0.0036 (12)	0.0076 (12)
C41	0.0672 (14)	0.0488 (12)	0.0929 (17)	−0.0070 (10)	0.0150 (12)	−0.0013 (11)
C51	0.0625 (13)	0.0426 (10)	0.0856 (16)	−0.0006 (9)	0.0168 (11)	0.0055 (10)
C52	0.0669 (14)	0.0478 (11)	0.0909 (17)	0.0106 (10)	0.0283 (12)	0.0071 (11)
C53	0.0870 (17)	0.0637 (14)	0.0839 (17)	0.0159 (13)	0.0157 (13)	0.0010 (13)
C54	0.111 (2)	0.087 (2)	0.092 (2)	0.0328 (17)	0.0149 (17)	−0.0087 (17)
C55	0.136 (3)	0.109 (3)	0.084 (2)	0.050 (2)	0.041 (2)	0.0164 (19)
C56	0.119 (2)	0.0805 (19)	0.111 (2)	0.0301 (18)	0.054 (2)	0.0272 (19)
C57	0.0857 (17)	0.0566 (13)	0.101 (2)	0.0170 (12)	0.0415 (15)	0.0166 (14)
C58	0.0781 (16)	0.0486 (12)	0.1046 (19)	−0.0086 (11)	0.0249 (14)	0.0076 (12)
N1	0.0576 (10)	0.0422 (8)	0.0795 (12)	−0.0059 (7)	0.0059 (8)	0.0044 (8)
N2	0.1034 (17)	0.0503 (11)	0.1236 (19)	−0.0162 (11)	0.0313 (14)	−0.0159 (12)
N3	0.0934 (15)	0.0496 (11)	0.1231 (19)	−0.0065 (10)	0.0430 (14)	0.0156 (12)
Cl1	0.1063 (6)	0.1147 (6)	0.0785 (5)	−0.0048 (4)	0.0132 (4)	0.0148 (4)

### Geometric parameters (Å, º)

**Table:**

C1—N1	1.340 (3)	C32—H32	0.9300
C1—C2	1.383 (3)	C33—C34	1.375 (3)
C1—C11	1.483 (3)	C33—H33	0.9300
C2—C3	1.385 (3)	C34—C35	1.377 (3)
C2—H2	0.9300	C34—Cl1	1.735 (3)
C3—C4	1.404 (3)	C35—C36	1.376 (3)
C3—C31	1.481 (3)	C35—H35	0.9300
C4—C5	1.410 (3)	C36—H36	0.9300
C4—C41	1.442 (3)	C41—N2	1.139 (3)
C5—N1	1.348 (3)	C51—C58	1.378 (3)
C5—C51	1.461 (3)	C51—C52	1.441 (3)
C11—C12	1.376 (3)	C52—C53	1.401 (4)
C11—C16	1.387 (3)	C52—C57	1.414 (3)
C12—C13	1.384 (3)	C53—C54	1.377 (4)
C12—H12	0.9300	C53—H53	0.9300
C13—C14	1.361 (4)	C54—C55	1.404 (5)
C13—H13	0.9300	C54—H54	0.9300
C14—C15	1.373 (4)	C55—C56	1.360 (5)
C14—H14	0.9300	C55—H55	0.9300
C15—C16	1.375 (3)	C56—C57	1.390 (4)
C15—H15	0.9300	C56—H56	0.9300
C16—H16	0.9300	C57—N3	1.367 (4)
C31—C36	1.389 (3)	C58—N3	1.344 (3)
C31—C32	1.399 (3)	C58—H58	0.9300
C32—C33	1.374 (3)	N3—H3	0.8600
			
N1—C1—C2	122.37 (18)	C32—C33—H33	120.2
N1—C1—C11	116.05 (19)	C34—C33—H33	120.2
C2—C1—C11	121.56 (18)	C33—C34—C35	120.8 (2)
C1—C2—C3	120.38 (19)	C33—C34—Cl1	120.2 (2)
C1—C2—H2	119.8	C35—C34—Cl1	119.0 (2)
C3—C2—H2	119.8	C36—C35—C34	119.5 (2)
C2—C3—C4	117.1 (2)	C36—C35—H35	120.2
C2—C3—C31	118.94 (19)	C34—C35—H35	120.2
C4—C3—C31	123.95 (19)	C35—C36—C31	121.2 (2)
C3—C4—C5	119.94 (18)	C35—C36—H36	119.4
C3—C4—C41	119.1 (2)	C31—C36—H36	119.4
C5—C4—C41	120.95 (19)	N2—C41—C4	179.6 (3)
N1—C5—C4	120.84 (18)	C58—C51—C52	106.1 (2)
N1—C5—C51	114.2 (2)	C58—C51—C5	128.2 (2)
C4—C5—C51	124.97 (18)	C52—C51—C5	125.68 (19)
C12—C11—C16	118.8 (2)	C53—C52—C57	118.2 (3)
C12—C11—C1	121.25 (19)	C53—C52—C51	135.5 (2)
C16—C11—C1	120.0 (2)	C57—C52—C51	106.3 (2)
C11—C12—C13	120.7 (2)	C54—C53—C52	119.4 (3)
C11—C12—H12	119.7	C54—C53—H53	120.3
C13—C12—H12	119.7	C52—C53—H53	120.3
C14—C13—C12	120.2 (3)	C53—C54—C55	121.0 (3)
C14—C13—H13	119.9	C53—C54—H54	119.5
C12—C13—H13	119.9	C55—C54—H54	119.5
C13—C14—C15	119.5 (2)	C56—C55—C54	120.9 (3)
C13—C14—H14	120.3	C56—C55—H55	119.5
C15—C14—H14	120.3	C54—C55—H55	119.5
C14—C15—C16	121.0 (3)	C55—C56—C57	118.5 (3)
C14—C15—H15	119.5	C55—C56—H56	120.7
C16—C15—H15	119.5	C57—C56—H56	120.7
C15—C16—C11	119.8 (3)	N3—C57—C56	130.5 (3)
C15—C16—H16	120.1	N3—C57—C52	107.6 (2)
C11—C16—H16	120.1	C56—C57—C52	121.9 (3)
C36—C31—C32	117.9 (2)	N3—C58—C51	110.1 (3)
C36—C31—C3	122.3 (2)	N3—C58—H58	124.9
C32—C31—C3	119.7 (2)	C51—C58—H58	124.9
C33—C32—C31	121.1 (2)	C1—N1—C5	119.22 (19)
C33—C32—H32	119.4	C58—N3—C57	109.9 (2)
C31—C32—H32	119.4	C58—N3—H3	125.0
C32—C33—C34	119.5 (2)	C57—N3—H3	125.0
			
N1—C1—C2—C3	−4.0 (3)	Cl1—C34—C35—C36	−178.62 (19)
C11—C1—C2—C3	177.71 (19)	C34—C35—C36—C31	0.1 (4)
C1—C2—C3—C4	1.8 (3)	C32—C31—C36—C35	−0.3 (3)
C1—C2—C3—C31	−178.89 (19)	C3—C31—C36—C35	−176.9 (2)
C2—C3—C4—C5	1.7 (3)	C3—C4—C41—N2	−129 (52)
C31—C3—C4—C5	−177.53 (19)	C5—C4—C41—N2	50 (52)
C2—C3—C4—C41	−179.1 (2)	N1—C5—C51—C58	157.8 (2)
C31—C3—C4—C41	1.7 (3)	C4—C5—C51—C58	−22.7 (4)
C3—C4—C5—N1	−3.4 (3)	N1—C5—C51—C52	−19.0 (3)
C41—C4—C5—N1	177.4 (2)	C4—C5—C51—C52	160.4 (2)
C3—C4—C5—C51	177.2 (2)	C58—C51—C52—C53	178.6 (2)
C41—C4—C5—C51	−2.0 (3)	C5—C51—C52—C53	−4.0 (4)
N1—C1—C11—C12	−17.6 (3)	C58—C51—C52—C57	0.1 (2)
C2—C1—C11—C12	160.8 (2)	C5—C51—C52—C57	177.5 (2)
N1—C1—C11—C16	161.9 (2)	C57—C52—C53—C54	−0.5 (3)
C2—C1—C11—C16	−19.7 (3)	C51—C52—C53—C54	−178.9 (2)
C16—C11—C12—C13	2.8 (4)	C52—C53—C54—C55	−0.4 (4)
C1—C11—C12—C13	−177.7 (2)	C53—C54—C55—C56	1.6 (4)
C11—C12—C13—C14	−0.7 (4)	C54—C55—C56—C57	−1.6 (4)
C12—C13—C14—C15	−2.3 (5)	C55—C56—C57—N3	179.5 (3)
C13—C14—C15—C16	3.1 (5)	C55—C56—C57—C52	0.7 (4)
C14—C15—C16—C11	−0.9 (5)	C53—C52—C57—N3	−178.6 (2)
C12—C11—C16—C15	−2.0 (4)	C51—C52—C57—N3	0.2 (2)
C1—C11—C16—C15	178.5 (3)	C53—C52—C57—C56	0.4 (3)
C2—C3—C31—C36	130.6 (2)	C51—C52—C57—C56	179.2 (2)
C4—C3—C31—C36	−50.2 (3)	C52—C51—C58—N3	−0.3 (3)
C2—C3—C31—C32	−46.0 (3)	C5—C51—C58—N3	−177.6 (2)
C4—C3—C31—C32	133.2 (2)	C2—C1—N1—C5	2.4 (3)
C36—C31—C32—C33	0.6 (3)	C11—C1—N1—C5	−179.26 (18)
C3—C31—C32—C33	177.3 (2)	C4—C5—N1—C1	1.3 (3)
C31—C32—C33—C34	−0.7 (4)	C51—C5—N1—C1	−179.23 (18)
C32—C33—C34—C35	0.4 (4)	C51—C58—N3—C57	0.4 (3)
C32—C33—C34—Cl1	178.91 (19)	C56—C57—N3—C58	−179.3 (3)
C33—C34—C35—C36	−0.1 (4)	C52—C57—N3—C58	−0.4 (3)

### Hydrogen-bond geometry (Å, º)

**Table:**

*D*—H···*A*	*D*—H	H···*A*	*D*···*A*	*D*—H···*A*
C58—H58···N2	0.93	2.58	3.285 (4)	133
N3—H3···N2^i^	0.86	2.20	3.037 (3)	164

Symmetry code: (i) −*x*, −*y*, −*z*.
