# Supplementary figures and images for: Crystal structure of 2-(4-chloro­phen­yl)-4-(1H-indol-3-yl)-6-phenyl­pyridine-3-carbo­nitrile
Source: Acta Crystallogr Sect E Struct Rep Online. 2014 Aug 6;70(Pt 9):o978. doi: 10.1107/S1600536814017693 (PMC4186124; doi:10.1107/S1600536814017693)

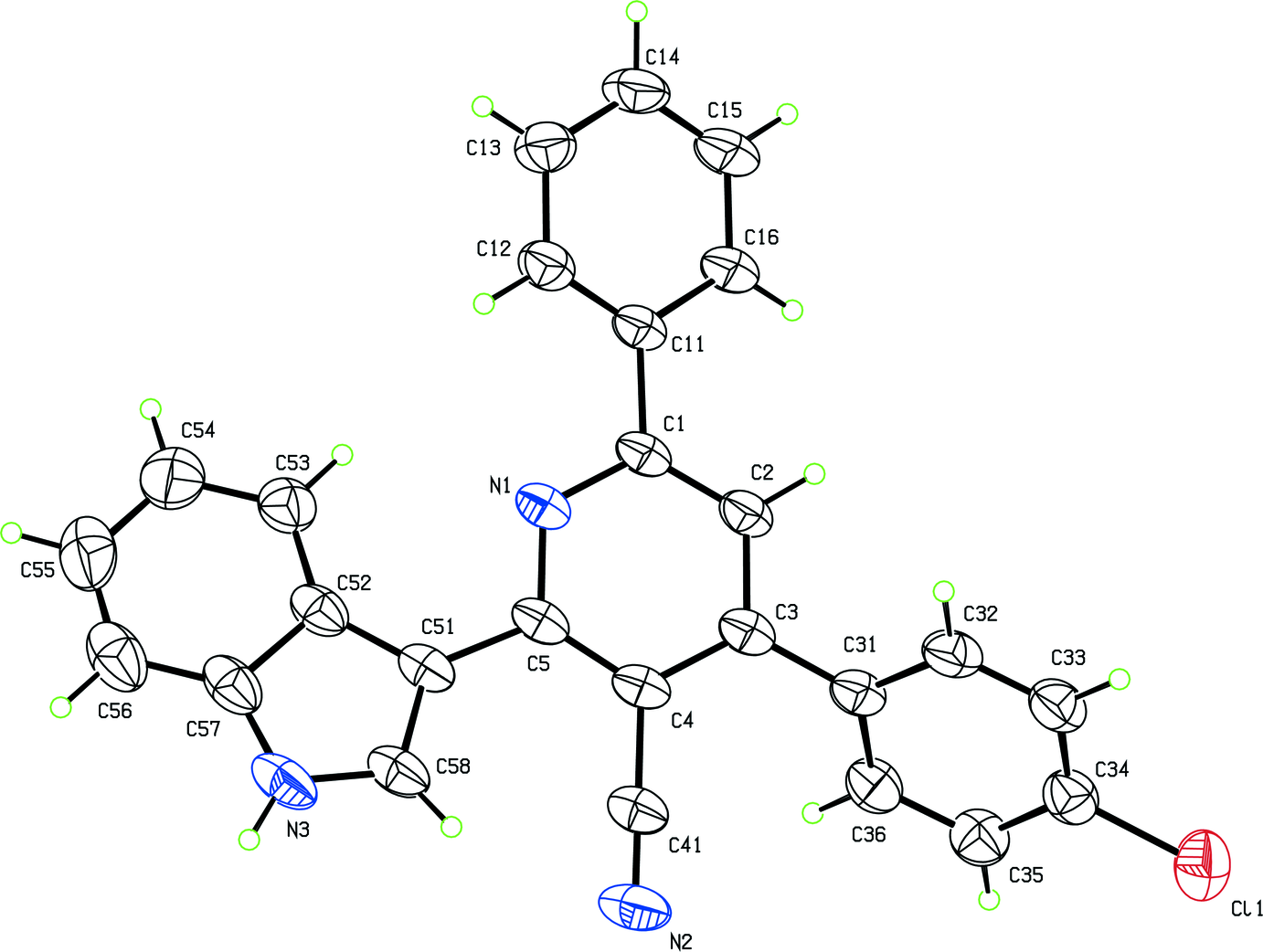

Supplement: Supplementary file 4 [file e-70-0o978-fig1.tif]

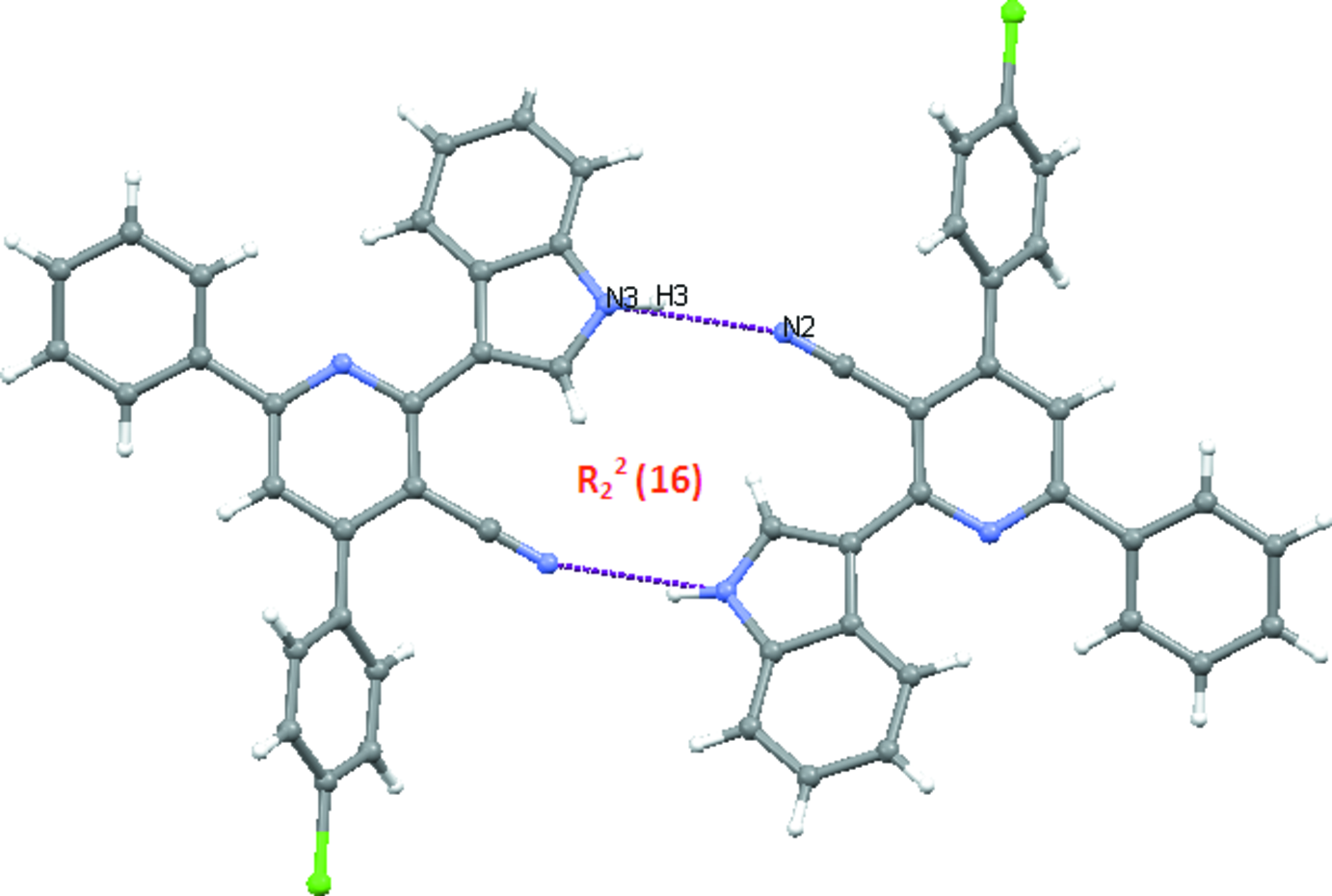

Supplement: Supplementary file 5 [file e-70-0o978-fig2.tif]
